# Danqi Pill Protects Against Heart Failure Post-Acute Myocardial Infarction *via* HIF-1α/PGC-1α Mediated Glucose Metabolism Pathway

**DOI:** 10.3389/fphar.2020.00458

**Published:** 2020-04-21

**Authors:** Qian Zhang, Dongqing Guo, Yuanyuan Wang, Xiaoping Wang, Qiyan Wang, Yan Wu, Chun Li, Wei Wang, Yong Wang

**Affiliations:** ^1^ School of Chinese Medicine, Beijing University of Chinese Medicine, Beijing, China; ^2^ The School of Life Sciences, Beijing University of Chinese Medicine, Beijing, China; ^3^ Institute of Chinese Medicine, Beijing University of Chinese Medicine, Beijing, China; ^4^ Modern Research Center for Traditional Chinese Medicine, Beijing University of Chinese Medicine, Beijing, China

**Keywords:** Danqi Pill (DQP), heart failure (HF), acute myocardial infarction (AMI), glucose metabolism, HIF-1α/PGC-1α pathway

## Abstract

**Aim:**

Heart failure (HF) post-acute myocardial infarction (AMI) leads to a large number of hospitalizations and deaths worldwide. Danqi pill (DQP) is included in the 2015 national pharmacopoeia and widely applied in the treatment of HF in clinics in China. We examined whether DQP acted on glucose metabolism to protect against HF post-AMI *via* hypoxia inducible factor-1 alpha (HIF-1α)/peroxisome proliferator-activated receptor α co-activator (PGC-1α) pathway.

**Methods and Results:**

In this study, left anterior descending (LAD) artery ligation induced HF post-AMI rats and oxygen-glucose deprivation-reperfusion (OGD/R)-induced H9C2 cell model were structured to explore the efficacy and mechanism of DQP. Here we showed that DQP protected the heart against ischemic damage as evidenced by improved cardiac functions and attenuated inflammatory infiltration. The expressions of critical proteins involved in glucose intake and transportation such as GLUT4 and PKM2 were up-regulated, while negative regulatory proteins involved in oxidative phosphorylation were attenuated in the treatment of DQP. Moreover, DQP up-regulated NRF1 and TFAM, promoted mitochondrial biogenesis and increased myocardial adenosine triphosphate (ATP) level. The protection effects of DQP were significantly compromised by HIF-1α siRNA, suggesting that HIF-1α signaling pathway was the potential target of DQP on HF post-AMI.

**Conclusions:**

DQP exhibits the efficacy to improve myocardial glucose metabolism, mitochondrial oxidative phosphorylation and biogenesis by regulating HIF-1α/PGC-1α signaling pathway in HF post-AMI rats.

## Introduction

Heart failure (HF) post-acute myocardial infarction (AMI) leads to a large number of hospitalizations and deaths worldwide (Ponikowski et al., 2016). Although the understanding of the mechanism of heart failure is deepening, new and different strategies for the treatment of HF are urgently needed.

Currently, one attractive approach for HF post-AMI treatment is to optimize myocardial substrate utilization (Torsten et al., 2013). Emerging evidence indicates that cardiomyocytes predominantly rely on glucose metabolism to produce ATP after AMI, which has higher energetic efficiency under ischemia (Nickel et al., 2013; Fillmore et al., 2014). Hence, focusing on glucose metabolism including glucose intake, transportation, and mitochondrial oxidation are extensively investigated (Diakos et al., 2016; Tuomainen and Tavi, 2017).

Due to hypoxia and ischemia after AMI, myocardial glucose intake and transportation were increased for compensatory ATP production (Brenner, 2018). Glucose transporter protein 4 (GLUT4), and pyruvate kinase M2 (PKM2) related to the myocardial glucose intake and metabolism pathway were activated (Szablewski, 2017; Williams et al., 2018). Furthermore, mitochondrial dysfunctions occur in the failing heart. The dysfunctions include impaired mitochondrial structure and electron transport chain components, inhibited oxidative phosphorylation, altered substrate utilization, increased ROS, and so on (Mori et al., 2012; Sang-Bing et al., 2013; Brown et al., 2017). Cardiac pyruvate dehydrogenase kinase 1 and 4 (PDK1 and PDK4) are downstream targets of peroxisome proliferator-activated receptor alpha (PPARα) (Czarnowska et al., 2016), which can phosphorylate and inhibit pyruvate dehydrogenase (PDH), the crucial enzyme catalyzing pyruvate to acetyl CoA to fuel mitochondrial TCA cycle (Fillmore and Lopaschuk, 2013). Uncoupling protein 2 (UCP2) is a mitochondrial proton carrier and functions in energy homeostasis (Akhmedov et al., 2015). Increased UCP2 provided channels for proton to transport into mitochondrial matrix, and then inhibited the ATP synthesis (Yang et al., 2018). Pecqueur C et al. show that UCP2 knocking-out cells preferentially utilize glucose metabolism instead of fatty acid oxidation (Pecqueur et al., 2008). Peroxisome proliferator-activated receptor co-activator α (PGC-1α) is a cofactor of PPARα transcription factors and their interaction regulates the expression of mitochondrial oxidation related genes (Scarpulla, 2011). PGC-1α also activates transcription factors nuclear respiratory factor 1 (NRF1) and mitochondrial transcription factor A (TFAM) to promote mitochondrial biogenesis and oxidation phosphorylation.

HIF-1α regulates the hypoxic response during an ischemic event (Sousa Fialho et al., 2018). HIF-1α is hydroxylated by prolyl hydroxylase 2 (PHD2) and the hydroxylated HIF-1α is easy to be degraded by ubiquitination proteasome pathway under normoxia (Wong et al., 2013). In hypoxia, accumulated HIF-1α is transported into the nucleus and form dimers with HIF-1β to regulated genes associated with glucose metabolism (Krishnan et al., 2009; Abud et al., 2012; Ambrose et al., 2014). This serves to increase intracellular glucose intake, augment glucose transportation, mitochondrial oxidative phosphorylation, and biogenesis (Hashmi and Al-Salam, 2012).

Danqi Pill (DQP) is a well-known Chinese patent medicine and used in treating coronary heart disease (Wang et al., 2016). However, the potential pharmacological mechanism of DQP on glucose metabolism remains unknown. Therefore, we examined whether DQP acted on glucose metabolism to protect against HF post-AMI *via* HIF-1α/PGC-1α pathway.

## Materials and Methods

### Drugs

Danqi Pill (DQP) is composed of two Chinese herbs at a composition of 1:1 (*Salvia miltiorrhiza* and *Panax notoginseng*). And the manufacturer is Beijing Tongrentang Pharmacy Co., Ltd. (Z11020471). The fingerprint of DQP was performed by high-performance liquid chromatography (HPLC) (**Figure S1**). Trimetazidine dihydrochloride Tablets (TMZ) were purchased from Servier Pharmaceutical Co., Ltd. (Tianjin, China, No. 2008344)

### Animals

Male Sprague-Dawley (SD) rats (220 g) were obtained from Beijing Vital River Laboratory Animal Technology Co., Ltd. Animal housing and experiments were under the guidelines for the Care and Use of laboratory animals (NIH), and the study ethically was approved by the Animal Care and Use Committee of Beijing University of Chinese Medicine. The acute myocardial infarction rat model was induced by left coronary artery anterior descending branch ligation as previously described (Wang et al., 2014). After the surgery, rats were randomly divided as sham, model, DQP-L (0.75 g/kg/day), DQP (1.5 g/kg/day), DQP-H (3.0 g/kg/day), and TMZ (6.3 mg/kg/day) group. Rats were treated with different drugs for 4 weeks.

### Echocardiography

All rats were anaesthetized using 1% pentobarbital sodium and subjected to echocardiographic examination. Echocardiography (Vevo 2100, Visual Sonics, Canada) was performed to assess the cardiac function. Parasternal short-axis M-mode frames were recorded and related parameters included left ventricular internal dimension-systole (LVID;s), left ventricular internal dimension-diastole (LVID;d), ejection fraction (EF), and fractional shortening (FS).

### Histological Examination

After 48 h fixing in the 4% paraformaldehyde, the myocardial tissues were embedded in paraffin and were sectioned at 5 μm thickness. Cardiac paraffin sections were stained with Hematoxylin-Eosin (HE) to evaluate the degree of inflammatory infiltration.

### Measurement of Myocardial ATP Levels

The fresh cardiac samples and cell lysates were prepared following the manufacture’s insturction of ATP assay kit (A095, Nanjing Jiancheng, China). ATP levels were detected and calculated in reference to the corresponding standard curves and were expressed as μmol/gprot.

### PET/CT Examination

Rats were in abrosia for 12 h before PET/CT scan. An amount of ~1 mCi of ^18^F-FDG was injected *via* the tail vein. After 20 min, PET-CT images were acquired (Inveon, Siemens Medical Solutions Knoxville, TN, United States). In our study, after being anesthetized with 1–1.5% isoflurane, the rats were placed in the supine position during PET/CT scanning and the acquisition time was 2~3 min per rats. Non-contrast-enhanced low-dose CT progressed by 30–80 kVp X-ray source.

Standardized uptake value (SUV) was calculated as follows: SUV=C/(D/M). C means activity concentration in the heart, D means injected dose, and M means body weight.

### Immunostaining Assay

Deparaffinized myocardial tissues sections (5 μm) were blocked with 5% sheep serum for 1 h and incubated with anti-GLUT4 (1:500) overnight at 4°C. After incubated with a secondary antibody, the samples were stained with diaminobenzidine (DAB) and re-stained with hematoxylin.

### Western Blotting Analysis of Protein Expressions

The proteins of heart tissues samples or H9C2 cells were extracted using RIPA lysis buffer containing a protease inhibitor. Protein samples were separated with 8%-10% sodium dodecyl sulfate polyacrylamide gel electrophoresis (SDS-PAGE) blots and then transferred to the PVDF membranes. These membranes were blocked with 5% non-fat milk for 1 h and then incubated with primary antibodies: anti-GLUT4 (#2213, CST, 1:2,000), anti-PKM2 (60268, Proteintech, 1:500), anti-NRF1 (12482-1, Proteintech, 1:500), anti-TFAM (22586-1, Proteintech, 1:500), anti-UCP2 (#89326s, CST, 1:200), anti-PDK4 (ab89295, Abcam, 1:1,000), anti-PDK1 (ab110025, Abcam, 1:1000), anti-PPARα (ab24509, Abcam, 1:2,000), anti-RXRα (#3085, CST, 1:1,000), anti-PGC-1α (ab54481, Abcam, 1:1,000), anti-HIF-1α (sc-10790, SANTA CRUZ, 1:500), anti-PHD2 (ab26058, Abcam, 1:1,000), anti-GAPDH (ab8245, Abcam, 1:5,000) at 4°C overnight. After three washes, the membranes were incubated with the secondary antibodies (goat anti-rabbit IgG 1:12,000 and goat anti-mouse IgG 1:5,000) for 1h. Proteins were detected using enhanced chemiluminescent (ECL) Plus Western blotting detection reagent (GE Healthcare, UK) by UVP BioImaging Systems (Bio-Rad, Hercules, CA, USA).

### Cell Culture and OGD/R Model

H9C2 cells were cultured in Dulbecco’s Modified Eagle Medium (DMEM) supplemented with 10% FBS and 1% penicillin/streptomycin under 5% CO_2_. In OGD/R model, cells were incubated using earle’s balanced salt solution and plated in a controlled hypoxic plastic chamber at 37°C for 8 h and then the cells were cultured in the normal medium and normoxia for 12 h. Cell viability was performed as previously described by CCK8 assay (Chang et al., 2017).

### Small Interfering RNA (siRNA) Transfection

H9C2 cells were transfected HIF-1α siRNA (50 nmol/L) using lipofectamine 2000^TM^ (Invitrogen). The sequences of HIF-1α siRNA were 5′GCAUUGAAGUUAGAGUCAAdTd3′, 3′UUGACUCUAACUUCAAUGCdTd5′, which were synthetized by Hanbio Biotechnology Co., Ltd. (Shanghai, China). After transfection, cells were randomly divided into control group, OGD/R group, OGD/R+DQP, OGD/R+HIF-1α siRNA+DQP, and OGD/R+HIF-1α siRNA treated group. The concentration of DQP on H9C2 cell was 400 μg/ml as described previously (Zhang et al., 2018).

### Mitochondrial Membrane Potential (△Ψm)

ΔΨm was assessed using the JC-1 staining kit (Beyotime Biotechnology, Shanghai, China). H9C2 cells were incubated with JC-1 probe for 20 min at 37°C. Cells were washed for three times with PBS and observed by a laser confocal microscopy (Leica Microsystems GmbH). The green fluorescence (Ex = 514 nm, Em = 529 nm) was used for monomers detection and the red fluorescence (Ex = 585 nm, Em = 590 nm) was used for J-aggregates detection. Five randomly chosen fields were photographed. The fluorescence intensity was analysed using Image ProPlus software and the ratio of aggregates/monomers fluorescence intensity was calculated.

### Statistical Analysis

Data was presented as mean ± SD. Analyses were performed by Graphpad 6 and SPSS 20.0 statistical software. One-way ANOVA were used to compare values between groups. P-value less than 0.05 was assumed to be statistical significance.

## Results

### DQP Reduced Cardiac Dysfunctions and Increased the ATP Level in HF Post-AMI Rats

**Figure 1:**
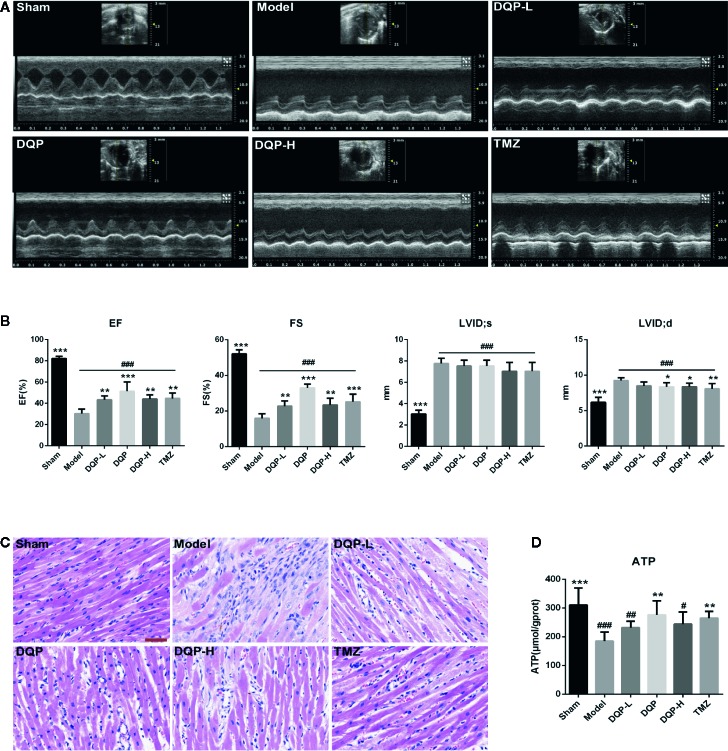
Danqi Pill (DQP) in different doses reduced cardiac dysfunctions, inhibited inflammatory cell infiltration and increased adenosine triphosphate (ATP) production. **(A)** Representative echocardiograms in heart sections. **(B)** Echocardiography analyses of ejection fraction (EF), fractional shortening (FS), left ventricular internal dimension-diastole (LVID;d), and left ventricular internal dimension-systole (LVID;s). **(C)** Histological analyses of H&E staining. The scale bar is 50 μm. **(D)** Myocardial ATP levels in different groups. ^#^ *P* < 0.05, ^##^ *P* < 0.01, **^###^***P*<0.001 compared with sham; ^*^ *P* < 0.05, ^**^ *P* < 0.01, ^***^ *P* < 0.001 compared with model. N = 6 per group.

Rat cardiac functions were evaluated by echocardiography (**Figure 1A**). Compared with model group, DQP with different doses could up-regulate EF and FS values significantly. LVID;d in DQP with different doses and TMZ group were decreased significantly while LVID;s in the four groups had no statistical differences, suggesting that DQP with different doses and TMZ improved cardiac function mainly through decreasing end-diastolic ventricular dilatation (**Figure 1B**). Histological evaluation of HE staining exhibited that treatment with DQP in three doses and TMZ attenuated inflammatory cell infiltration and edema of cardiomyocytes caused by ischemia (**Figure 1C**). Energy in the form of ATP is needed for the heart to fuel its contractile machinery and the ionic pumps that serve to regulate its function (Kadkhodayan et al., 2013). DQP and TMZ treatment increased myocardial ATP levels significantly (**Figure 1D**). These data implicate that DQP could rescue heart function in HF post-AMI rats.

### DQP Promoted Cardiac Glucose Metabolism in HF Post-AMI Rats

**Figure 2:**
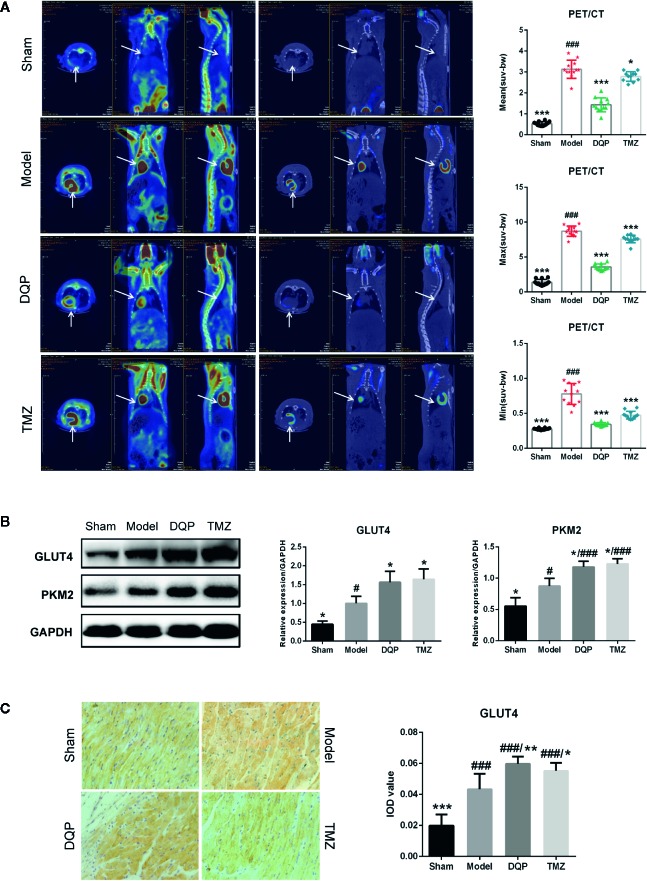
Danqi Pill (DQP) promoted cardiac glucose metabolism in heart failure (HF) post-acute myocardial infarction (AMI) rats. **(A)** ^18^F-FDG uptake detected by PET/CT in four groups. Quantitative analyses of mean, max, and min standardized uptake value (SUV). **(B)** Cardiac protein expressions of GLUT4 and PKM2 and densitometric analyses. **(C)** Immunostaining of GLUT4 in cardiac sections and quantification of GLUT4. Region of interest (ROI) = 12 per rat. **^#^***P* < 0.05, **^###^***P* < 0.001 compared with sham; ^*^P < 0.05, ^**^P < 0.01, ^***^P < 0.001 compared with model. N = 3 per group for western blotting. N = 6 per group for IHC.

It has been widely reported that substrate utilization change in the heart failure and metabolic therapy has attracted widespread attention (Stanley et al., 2005). Fatty acid oxidation is the main fuel source for normal cardiomyocytes, however it shifts to glucose metabolism in the process of HF (Brown et al., 2017). To elucidate the effects of DQP on glucose metabolism, PET/CT was firstly applied to assess the uptake of glucose located in myocardial tissue. PET/CT images exhibited that abnomal accumulation of ^18^F-FDG in the heart of HF post-AMI rat, which may be caused by increased inflammatory response and glucose metabolism disorders (Aoyama et al., 2017; Wisenberg et al., 2019). DQP and TMZ could significantly reduce the accumulation of glucose in the rat heart (**Figure 2A**). GLUT4 is the main protein to assist glucose transport (Vargas et al., 2019), while PKM2 is the key enzyme involved in the glucose metabolism (Zhang et al., 2019). Therefore, we explored whether DQP influenced the expression of pivotal proteins involved in the glucose intake and metabolism. DQP treatment could promote GLUT4 and PKM2 expressions compared with HF post-AMI rats determined by Western blots (**Figure 2B**). Histological results also revealed that DQP and TMZ both increased the GLUT4 expression (**Figure 2C**).

### DQP Promoted Glucose Metabolism by Activating Myocardial Mitochondrial Oxidation Pathway in HF Post-AMI Rats

**Figure 3:**
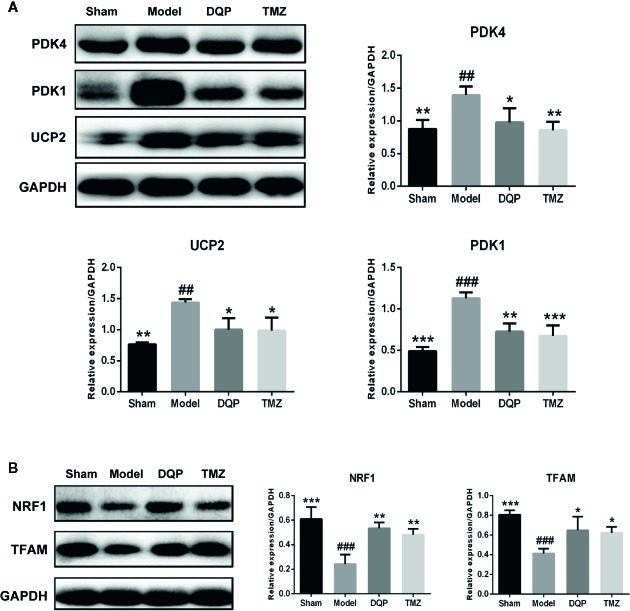
Danqi Pill (DQP) promoted glucose metabolism by activating myocardial mitochondrial oxidation pathway in heart failure (HF) post-acute myocardial infarction (AMI) rats. **(A)** Western blot analysis the expressions of PDK4, PDK1, and UCP2 in all groups. **(B)** Western blot analysis the expressions of NRF1 and mitochondrial transcription factor A (TFAM) in all groups. Densitometric analysis was shown in the graph. GADPH was used as internal reference. **^##^***P* < 0.01, **^###^***P* < 0.001 compared with sham; ^*^P < 0.05, ^**^P < 0.01,^***^P < 0.001 compared with model. N = 3 per group.

The heart is a high energy consuming organ, which needs to consume a lot of ATP to maintain its contraction. In addition to fatty acid oxidation, glucose aerobic oxidation is another important energy source for the heart (van Bilsen et al., 2004). UCP2 and two subtypes of PDKs named as PDK4 and PDK1 were chosen to assess the ability of mitochondrial oxidative phosphorylation. As shown in **Figure 3A**, the expressions of PDK4, PDK1, and UCP2 in model group were significantly increased by 37.25%, 56.53% and 46.83% compared with the sham group. In DQP treated group compared with model group, expressions of PDK4, PDK1, and UCP2 were decreased respectively by 29.92%, 35.61%, and 30.34%. Then, we detected the proteins involved in the mitochondrial biogenesis including NRF1 and TFAM. DQP and TMZ treatment significantly promoted the protein production of NRF1 and TFAM (**Figure 3B**).

### DQP Activated HIF-1α/PGC-1α Signaling Pathway in HF Post-AMI Rats

**Figure 4:**
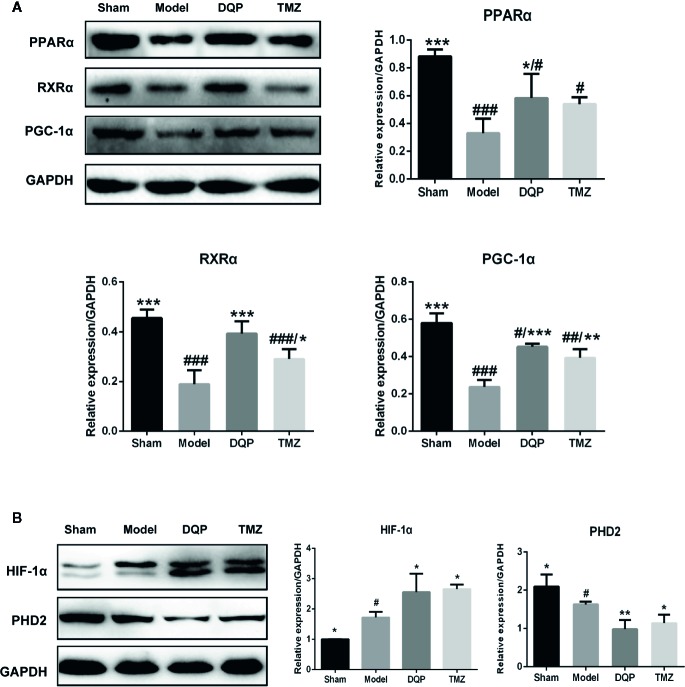
Danqi Pill (DQP) activated HIF1α/PGC1α signaling pathway in heart failure (HF) post-acute myocardial infarction (AMI) rats. **(A)** Western blot analysis the expressions of PPARα, RXRα, and PGC-1α in the four groups. Densitometric analysis was showed. **(B)** Effects of DQP on expressions of HIF-1α and PHD2 in HF post-AMI Rats; WB bands and protein quantitative results of HIF-1α and PHD2 in heart tissues of rats. ^#^ *p* < 0.05, ^##^ *p* < 0.01, **^###^***P* < 0.001 compared with sham; ^*^P < 0.05, ^**^P < 0.01, ^***^P < 0.001 compared with model. N =3 per group.

Extensive research indicates that PGC-1α interacts with other transcription factors, such as RXRα and PPARα to regulate the mitochondrial biogenesis and oxidative phosphorylation (Duncan, 2011; Wang et al., 2013). DQP treatment could increase PPARα, RXRα, and PGC-1α protein levels by 77.02%, 107.64%, and 90.53% compared with model rats, respectively (*P*<0.001). TMZ had similar effects as DQP, but the promotion effects of TMZ were milder than DQP (*P*<0.05, **Figure 4A**). HIF-1α plays a key role in hypoxia adaptation by promoting the expression of hypoxia inducible genes and participating in the specific response of tissue cells to hypoxia (Suzuki et al., 2017). DQP and TMZ treatment dramatically increased HIF-1α protein level in the heart of HF post-AMI rats, as determined by western blots. PHD2 acts as the primary rate-limiting HIF prolyl hydroxlase which affects transcriptional stability of HIF-1α (Flashman et al., 2010). DQP and TMZ could markedly downregulated the expressions of PHD2 respectively (**Figure 4B**).

### DQP Protected Against OGD/R-Induced H9C2 Cells Injury *via* Regulating HIF-1α

**Figure 5:**
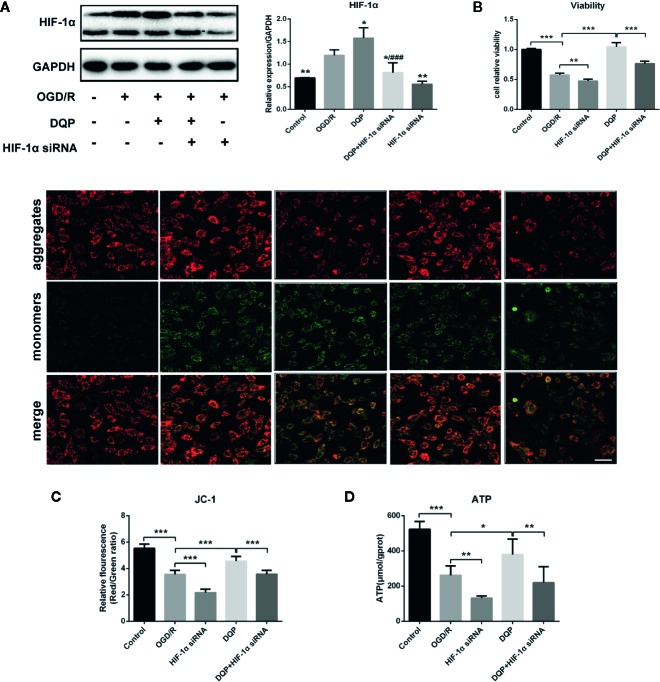
Danqi Pill (DQP) protected against oxygen-glucose deprivation-reperfusion (OGD/R)-induced H9C2 cells injury *via* up-regulating HIF-1α. **(A)** Immunoblot of HIF-1α in H9C2 Cells. **(B)** HIF-1α small interfering RNA (siRNA) suppressed the facilitation of DQP in the proliferation of OGD/R H9C2 cells. **(C)** HIF-1α siRNA decreased mitochondrial membrane potential (△Ψm) with/without DQP treatment in OGD/R-induced H9C2 cells. △Ψm was measured by JC-1 probe (400 ×, Scale bar 100 μm). **(D)** Intracellular adenosine triphosphate (ATP) levels in H9C2 Cells. ^*^ *P* < 0.05, ^**^ *P* < 0.01, ^***^ *P* < 0.01 compared with model; ^###^ *P* < 0.001 compared with DQP. N = 3 per group for WB and N=6 for the other experiments.

To determine the role of HIF-1α on the protection of DQP in OGD/R-induced H9C2 cells, HIF-1α siRNA was applied. Immunoblots showed that OGD/R could induce HIF-1α production, whereas DQP treatment further promoted the increase of HIF-1α. In our study, siRNA was applied to interfere in HIF-1α expression, while DQP could increase HIF-1α level in H9C2 transfected with siRNA (**Figure 5A**). CCK-8 assay results demonstrated HIF-1α siRNA could suppress the facilitation of DQP in the proliferation of OGD/R H9C2 cells (**Figure 5B**). The assessment of mitochondrial transmembrane potential was performed by JC-1 probe. The findings showed that the ratio of aggregates/monomers increased in response to DQP, suggesting the △Ψm returned to normal. However, the ratio decreased when using HIF-1α siRNA (**Figure 5C**). Intracellular ATP concentration also was examined in OGD/R-induced H9C2 cells. ATP level was up-regulated in response to DQP, while it was neutralized by using HIF-1α siRNA (**Figure 5D**). Overall, the protective mechanism of DQP was related to HIF-1α pathway.

## Discussion

In the current study, we investigated the pharmacological mechanisms of DQP on glucose metabolism *via* the HIF-1α/PGC-1α signaling pathway. The results indicated that: (1) DQP inhibited cardiac dysfunction and inflammatory infiltration, increased ATP levels in the HF post-AMI rats. (2) DQP improved glucose metabolism through myocardial glucose intake and transportation pathway in HF post-AMI rats. (3) DQP treatment promoted mitochondrial oxidation phosphorylation and mitochondrial biogenesis. (4) The protective effects of DQP were associated with HIF-1α/PGC-1α pathway.

Heart contractions require a great deal of ATP, which is mainly from myocardial fatty acid oxidation and glucose metabolism (Niemann et al., 2018). However, the failing heart predominantly relies on anaerobic glycolysis to produce ATP after AMI, and decreases the use of glucose oxidation metabolism (Karwi et al., 2018). In our previous study, DQP treatment increased local oxygen supply by inhibiting the damage of cardiomyocytes and promoting angiogenesis (Jiao et al., 2018), accompanying with the increasing lipid metabolism (Zhang et al., 2018). Increased angiogenesis and oxygen supply also promote glucose oxidation. The essential process of glucose aerobic oxidation is the glycolytic pathway in which GLUT4 and PKM2 play essential roles. The expressions of GLUT4 and PKM2 in ischemic myocardial tissue were promoted to ensure the energy supply after the intervention of DQP.

HIF-1α could increase myocardial glucose intake and transportation in order to continuously provide the compensatory energy supply by regulating myocardial GLUT4 and PKM2 genes expressions (Rees et al., 2015). HIF-1α also facilitates activation of PDK1 and PDK4 as well as UCP2 to enhance the mitochondrial oxidative phosphorylation (Cunha-Oliveira et al., 2018). Moreover, NRF1 and TFAM play distinct roles in mitochondrial biogenesis (Yao et al., 2016) and the upregulation of NRF1 and TFAM promotes mitochondrial DNA synthesis in infarcted cardiac muscle (Sheeran and Pepe, 2017). Therefore, HIF-1α signaling pathway is activated in cardiomyocytes to produce continuous ATP in adaption to hypoxia, by shifting myocardial metabolism substrate to glucose intake and transportation (Karwi et al., 2018). The levels of PDK1, PDK4, and UCP2 were weakened in the failing heart with DQP treatment, suggesting that DQP functioned by promoting glucose intake, transportation and oxidative phosphorylation to produce more ATP. Moreover, DQP could upregulate NRF1 and TFAM to promote the mitochondrial biogenesis. The up-stream pathways involved in regulating myocardial mitochondrial oxidation, glucose intake and transportation were further investigated. DQP could promote the expressions of PPARα, RXRα, PGC-1α, and HIF-1α. To further identify the effect of DQP on HIF-1α, the oxygen-dependent regulatory hydroxylases PHD2, which manifested affinity and specificity for each HIF-1α forming a feedback loop suffered ischemia (He et al., 2018), was investigated. Consistently, DQP promoted HIF-1α expression and weakened PHD2 expression. Moreover, HIF-1α siRNA abolished the protective effect of DQP on energy metabolism demonstrating that DQP could partly activate the HIF-1α and exert myocardial protection through HIF-1α signaling pathway.

As the limitations of our study, the active components of DQP participated in regulating HIF-1α/PGC-1α mediated glucose metabolism pathway are still unknown. We will further explore the combination of active components and HIF-1 α targets in DQP, so as to explain the regulatory mechanism of DQP more systematically and comprehensively. Besides, we found that different ligation sites might lead to different effects on the cardiac function and more experiments need to be conducted to confirm it in the future study.

## Conclusion

**Figure 6:**
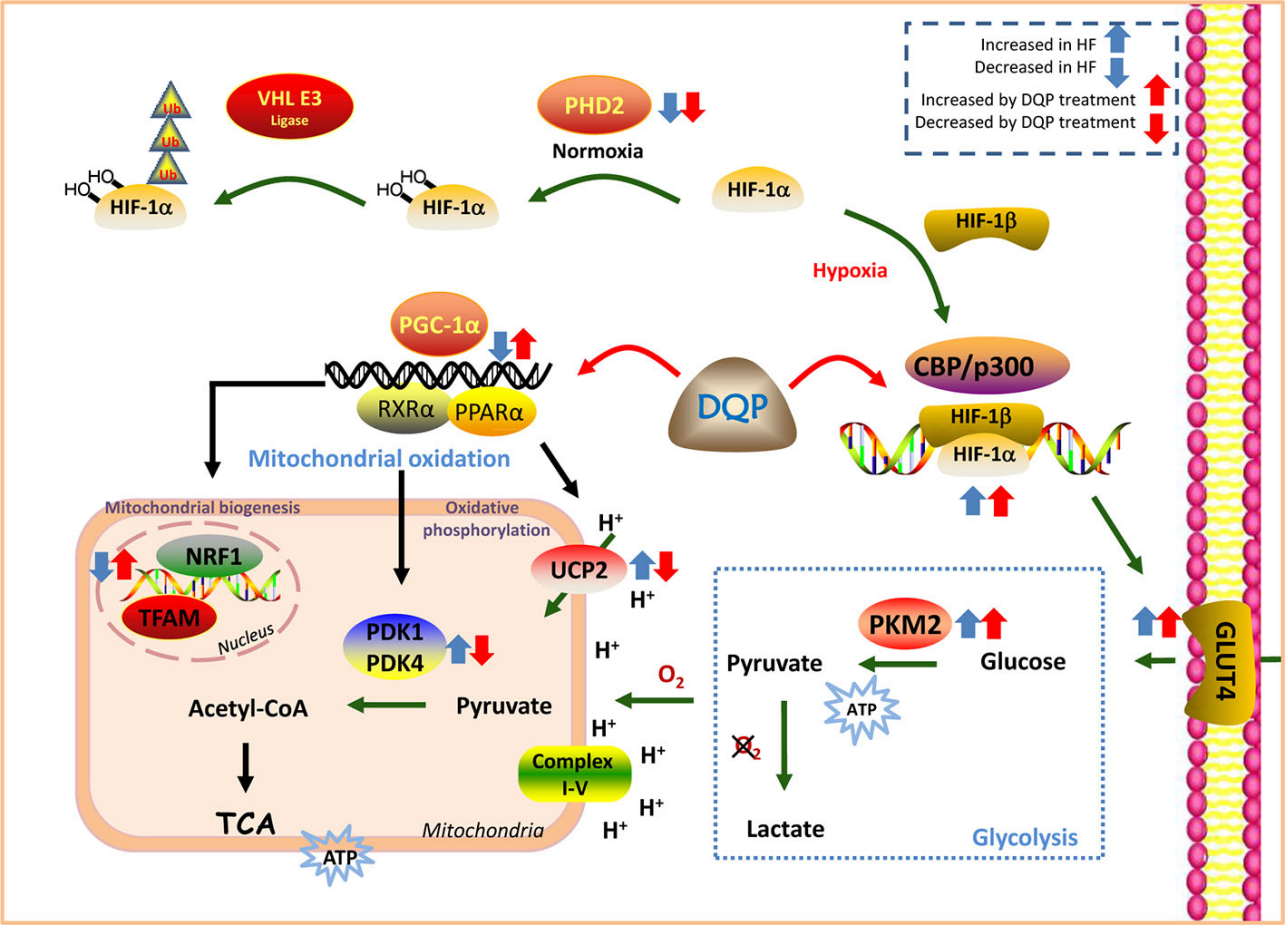
Danqi Pill (DQP) targeted on HIF-1α/PGC-1α signaling pathway and finally elevated cardiac energy metabolism in the failing heart.

DQP has the efficacy to improve myocardial glucose intake, transportation and mitochondrial biogenesis and oxidative phosphorylation both *in vivo* and *in vitro*. The effects may be mediated by regulation of HIF-1α/PGC-1α signaling pathway (**Figure 6**).

## Data Availability Statement

All datasets generated for this study are included in the article/**Supplemental Material**.

## Ethics Statement

The animal study was reviewed and approved by the Animal Care and Use Committee of Beijing University of Chinese Medicine.

## Author Contributions 

CL, WW, and YoW conceived and designed the project. Each author has contributed significantly to the submitted work. QZ, DG, and YyW performed the research, analyzed the data, and drafted the manuscript. XW, QW, and YaW revised the manuscript. All authors read and approved the final manuscript.

## Funding

This work was financially supported, in part, by grants from the National Natural Science Foundation of China (No. 81822049, 81673712, and 81673802), the New drug creation of Ministry of science and technology (No. 2019ZX09201004-001-011), the Fok Ying Tung Education Foundation (No. 151044), the Beijing Nova Program (Z171100001117028), and the Talent Young Scientist of China Association for Science and Technology (No. CACM-2017-QNRC2-C13, No. CACM-2018-QNRC2-C07).

## Conflict of Interest

The authors declare that the research was conducted in the absence of any commercial or financial relationships that could be construed as a potential conflict of interest.
