# Supplementary figures and images for: Danqi Pill Protects Against Heart Failure Post-Acute Myocardial Infarction via HIF-1α/PGC-1α Mediated Glucose Metabolism Pathway
Source: Front Pharmacol. 2020 Apr 21;11:458. doi: 10.3389/fphar.2020.00458 (PMC7187888; doi:10.3389/fphar.2020.00458)

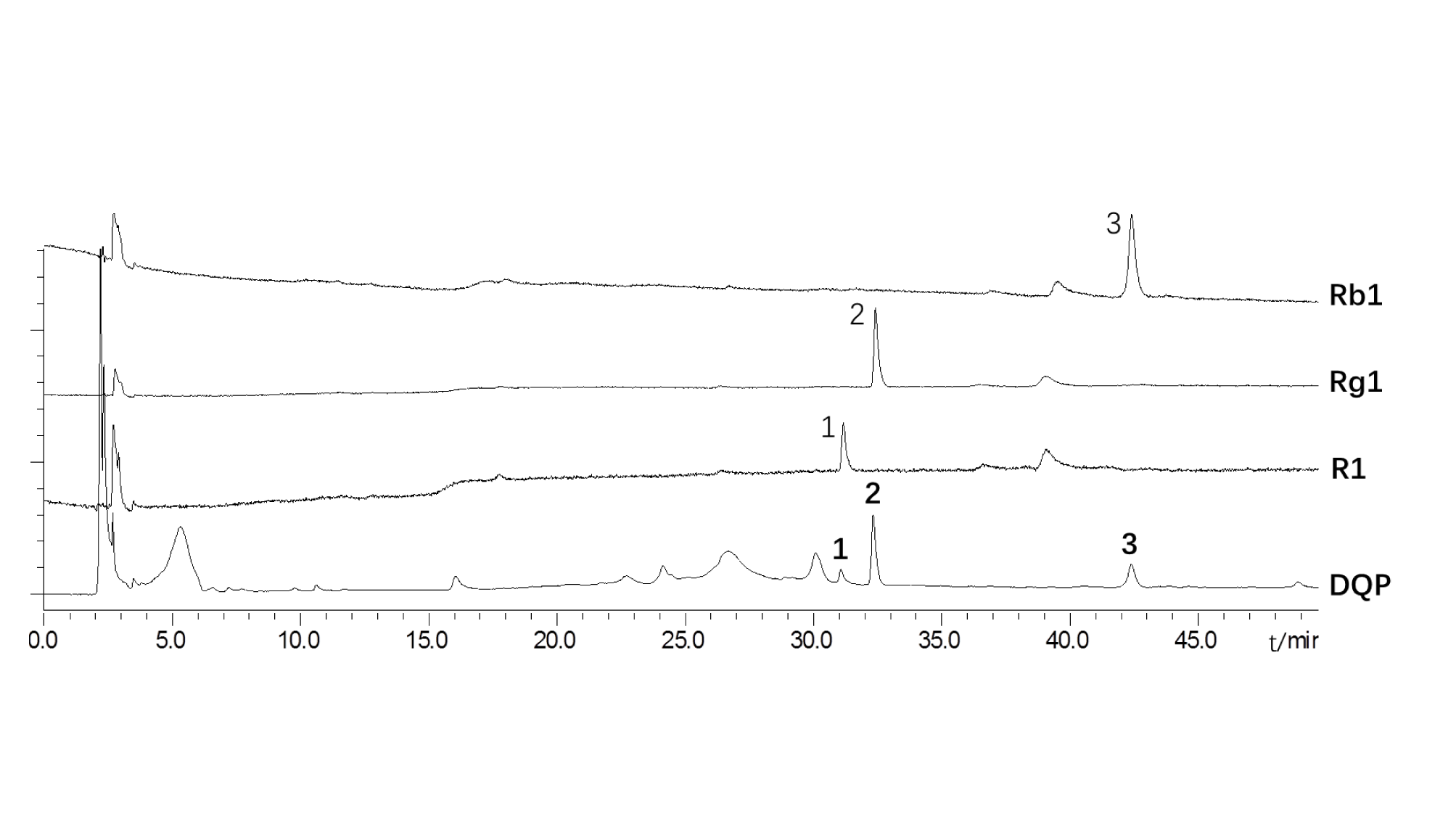

Supplement: Supplementary file 1 [file Image_1.tif]
